# Sediment Composition Influences Spatial Variation in the Abundance of Human Pathogen Indicator Bacteria within an Estuarine Environment

**DOI:** 10.1371/journal.pone.0112951

**Published:** 2014-11-14

**Authors:** Tracy L. Perkins, Katie Clements, Jaco H. Baas, Colin F. Jago, Davey L. Jones, Shelagh K. Malham, James E. McDonald

**Affiliations:** 1 School of Biological Sciences, Bangor University, Bangor, United Kingdom; 2 School of Ocean Sciences, Bangor University, Bangor, United Kingdom; 3 School of Environment, Natural Resources and Geography, Bangor University, Bangor, United Kingdom; 4 Centre for Applied Marine Sciences, Bangor University, Bangor, United Kingdom; University of Aveiro, Portugal

## Abstract

Faecal contamination of estuarine and coastal waters can pose a risk to human health, particularly in areas used for shellfish production or recreation. Routine microbiological water quality testing highlights areas of faecal indicator bacteria (FIB) contamination within the water column, but fails to consider the abundance of FIB in sediments, which under certain hydrodynamic conditions can become resuspended. Sediments can enhance the survival of FIB in estuarine environments, but the influence of sediment composition on the ecology and abundance of FIB is poorly understood. To determine the relationship between sediment composition (grain size and organic matter) and the abundance of pathogen indicator bacteria (PIB), sediments were collected from four transverse transects of the Conwy estuary, UK. The abundance of culturable *Escherichia coli*, total coliforms, enterococci, *Campylobacter*, *Salmonella* and *Vibrio* spp. in sediments was determined in relation to sediment grain size, organic matter content, salinity, depth and temperature. Sediments that contained higher proportions of silt and/or clay and associated organic matter content showed significant positive correlations with the abundance of PIB. Furthermore, the abundance of each bacterial group was positively correlated with the presence of all other groups enumerated. *Campylobacter* spp. were not isolated from estuarine sediments. Comparisons of the number of culturable *E. coli*, total coliforms and *Vibrio* spp. in sediments and the water column revealed that their abundance was 281, 433 and 58-fold greater in sediments (colony forming units (CFU)/100****g) when compared with the water column (CFU/100****ml), respectively. These data provide important insights into sediment compositions that promote the abundance of PIB in estuarine environments, with important implications for the modelling and prediction of public health risk based on sediment resuspension and transport.

## Introduction

Estuarine environments represent some of the most biologically productive systems in the biosphere and consequently provide a wealth of economic, social and natural ecosystem services that include food, employment, recreation and habitat [1]. However, the sustainability of such systems can be severely compromised along developed and urbanised coastlines, and this is predominantly due to anthropogenic influences [2]. Almost half of the world’s population are thought to live within a few hundred kilometers of the coast [3] and consequently, anthropogenic activities have a significant influence upon the health of estuarine and ocean ecosystems. Furthermore, as the global climate changes, meteorological events such as storms and floods present further impacts upon estuarine environments, and increased rainfall will significantly impact the flow and transportation of microbial pollution from the terrestrial environment into the coastal zone [4]. Human pathogenic microorganisms are introduced into estuarine ecosystems via the release of effluent from wastewater treatment plants, ineffective septic tank systems and storm water runoff [5]. Agricultural runoff from livestock farming can also represent a major source of microbial pollution, particularly when excreted waste from poultry and livestock is re-applied to land [6]. Wildlife, especially migratory wildfowl and other birds also represent an important source of zoonotic bacterial pathogens in natural environments [7].

Human pathogenic bacteria often occur at low levels in the environment [8] and their specific detection is a laborious and costly process [9]. Consequently, the detection and enumeration of faecal indicator bacteria that are present in the gastrointestinal tract of warm-blooded animals in much higher quantities represent a more effective ‘indicator’ of risk [10]. Current legislation on water quality testing relies on the enumeration of faecal coliforms (including the well-characterised *E. coli*) and enterococci to assess and classify water quality [11]. However, several other groups of potentially pathogenic bacteria have been detected in estuarine and marine environments. These include *Salmonella* spp. [12], *Campylobacter* spp. [13] and *Vibrio* spp. [14]. *Salmonella* spp. are well-known foodborne pathogens and recognised as one of the main causes of gastroenteritis in humans [15], resulting in around 1.3 billion annual cases of infection worldwide [16] but their presence in bathing waters is not routinely monitored [17]. Pathogenic strains of *Campylobacter* spp. are also thought to be responsible for a large proportion of enteric illnesses in humans living in developed and industrialized countries [18]. Contamination of surface and coastal waters by *Salmonella* and *Campylobacter* spp. is thought to occur predominantly via faecal contamination from wildfowl and other birds, although other animals including domestic livestock are also reservoirs [19]. *Vibrio* spp. are ubiquitous in aquatic environments and some strains are pathogens of humans; *V*. *cholera* has been responsible for several previous pandemics, resulting in high human mortality rates [20], some of which originated from the consumption of contaminated seafood [21]. Consequently, filter-feeding shellfish such as mussels, scallops and oysters are especially susceptible to contamination with bacterial pathogens if grown in contaminated waters [22].

Spatial and temporal variations in bacterial abundance in an environment are controlled by the interactions of complex physical, chemical and biological parameters, such as available nutrients [23], organic matter [24], sediment grain size [25], clay content [26], heavy metal content [27], predation by protozoa [28], competition [29], temperature [30], salinity [31], sunlight intensity [32] and seasonal variations [33]. Consequently, association with particulate material and sediments offers several advantages in terms of the survival and persistence of bacterial pathogens, such as protection from UV light [34], [35], protection from phage attack in saline conditions [36], shelter from predation [37] and a greater organic matter content compared to the water column [38]. However, hydrodynamic processes (e.g. tides and storms), recreational activities and mechanical disturbances such as commercial dredging all have the potential to re-suspend sediment particles and their associated pathogens back into the water column, resulting in periodic elevated levels of pollution [39], [40]. Currently, classification of both bathing and shellfish water quality relies solely upon the enumeration of FIB in water samples, despite the well-established paradigm that allochthonous bacterial pathogens in the water column become preferentially attached to particulate material, which promotes their downward flux to the bottom sediments where they are typically found in greater abundance [41], [42].

The Conwy catchment, North Wales, UK, has a population of approximately 112,000, 80% of which live in coastal resorts that represent the main economic and tourist areas, with over 5.4 million visitors per annum. The Conwy estuary directly impacts commercially important shellfish beds, bathing waters and beaches; hence deterioration of the water quality could have major socioeconomic consequences. The catchment covers an area of ∼300****km^2^ which consists of large areas of land utilised for agriculture [43], some of the highest mountains in the UK [44], in addition to residential and commercial areas. Consequently, the Conwy river and it’s tributaries (such as the river Gyffin and river Ganol) receive wastewater effluent from several waste water treatment plants in addition to septic tank discharges. Microbiological inputs from wildlife, agriculture, wastewater effluent, septic tank discharges and run-off from storm events therefore represent potential point and diffuse sources of FIB in the Conwy estuary.

Previous analysis of FIB concentrations in the water column across four transverse transects of the Conwy estuary revealed significant spatial variations in waterborne *E. coli* numbers on contrasting sides of the estuary (45). These data suggested that significantly different levels of microbial pollution were present in the east and west sides of the river (45) and consequently, the choice of sampling points for routine water quality monitoring could have a significant impact upon the classification of microbiological water quality in this area. The Conwy estuary has a dynamic tidal cycle, with resuspension and deposition of finer sediment particles observed on the shallower banks of the river, resulting in the formation of mud flats. Consequently, the localised resuspension of sediment associated FIB into the water column was one proposed explanation for such contrasting FIB counts in the water column of the Conwy estuary (45), yet the spatial relationships between sediments composition (grain size and organic matter content) and FIB abundance in the Conwy and beyond has received little attention.

Whilst most studies focus on enumeration of *E. coli* and enterococci in water samples for the assessment of public health risk, the aim of our study was to address the paucity of information regarding the ecology of FIB along with other potentially human-pathogenic bacteria in estuarine sediments, and to identify sediment characteristics that promote pathogen abundance in estuarine environments. A second aim was to determine if the enumeration of *E. coli,* coliforms and enterococci, that represent the current standard ‘indicator’ organisms for faecal contamination and water quality monitoring, are a suitable indicator for the co-occurence of other potentially pathogenic bacterial groups. This was achieved by determining spatial variations in the abundance of culturable *E. coli*, total coliforms, enterococci, *Salmonella*, *Campylobacter* and *Vibrio* spp. (to be used as a proxy for potential human bacterial pathogens in the estuary and referred to as ‘pathogen indicator bacteria’ (PIB) herein) in estuarine sediments of the Conwy Estuary, UK, in relation to sediment grain size, organic content and other physico-chemical factors.

## Materials and Methods

### Study site

**Figure 1:**
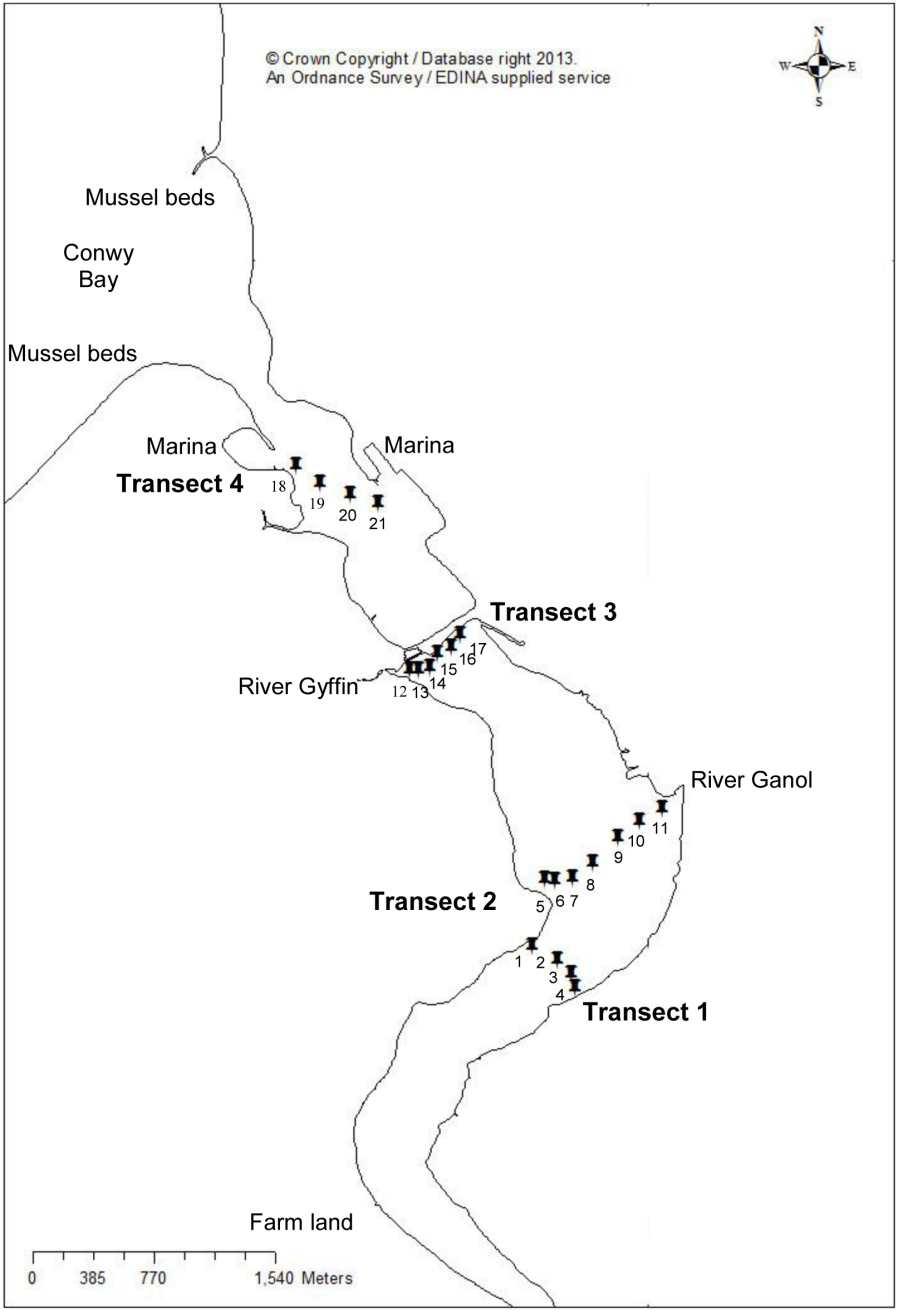
A map of the study site; the Conwy Estuary, North Wales, UK. Water and sediment samples were collected in triplicate from four transverse transects of the Conwy estuary (twenty one sampling sites).

Sample locations were selected to correspond to those tested in a previous study that observed spatial variations of waterborne *E. coli* within the study area [45], in addition to possible point and diffuse sources of pollution that include mudflats used by wading birds and agricultural land (transect 1), input from the river Ganol (transect 2), the river Gyffin (transect 3) and Conwy marina (transect 4), (Figure 1).

### Estuarine sampling

Estuarine sediment and water samples were collected from four transverse transects of the estuary over two consecutive sampling days (transects 1 and 2 collected on the 14/05/12, transects 3 and 4 on the 15/05/12). The Bangor University research vessel “Macoma”, a 7.9 m Cheetah catamaran survey boat was utilised for sample collection, which was initiated at slack water. For each sampling point, three replicate sediment samples were collected using a manually operated Van Veen sediment grab, approximately 50 g of bottom sediment from the centre of each grab was transferred into sterile 50****ml polypropylene tubes (VWR International Ltd., Leicestershire, UK), water samples were collected in triplicate from 0.2****m below the water surface using a sterile 1 L polypropylene container, then transported to the laboratory where water samples were processed within 4 h and sediment samples within 6 h.

### Isolation and cultivation of target bacterial groups from sediment

One gram of each sediment sample was transferred to a 7****ml sterile bijou tube (Starlab UK Ltd., Milton Keynes, UK) and suspended in 5****ml Ringers solution (Oxoid Ltd., Basingstoke, UK) to obtain a 1∶5 (w/v) dilution. Each sample was vortexed for 90****s to disassociate and resuspend bacteria from the sediment. Aliquots of the resulting supernatant for each sample were transferred aseptically onto agar plates containing a selective medium for *E. coli*, total coliforms, enterococci, *Vibrio* spp., *Campylobacter* spp., *Salmonella* spp. and total heterotrophs. The optimum volume of supernatant used to inoculate each selective medium was determined in a previous study (data not shown). All selective medium plates were inverted and incubated according to manufacturer’s recommendations. Resulting colony forming units (CFUs) provided enumeration of bacterial groups. Details describing the selective media used and incubation times are described in Table S1 in the supplemental material. The CFU data for sample point 13 represents the average of only two sediment samples due to one of the replicates being lost as a result of the presence of gravel clasts. All other CFU data represent the average of three independent replicate samples.

### Isolation and cultivation of target bacterial groups from water

Water samples were processed within 4****h in accordance with the Revised Bathing Water Directive (2006/7/EC). Enumeration of bacteria in water samples was achieved by using vacuum–filtration as described in [45]. Briefly, water samples were homogenised by shaking and 50****ml of water filtered under vacuum through a 0.2****µm cellulose acetate membrane (Sartorius Stedim Biotech., Gottingen, Germany). Subsequently, the membranes were aseptically transferred onto sterile agar plates containing selective medium for the enumeration of *E. coli*, total coliforms, *Vibrio* spp. and heterotrophic bacteria. Agar plates were inverted and incubated according to manufacturer’s recommendations (Table S1). Resulting Colony Forming Units (CFUs) were enumerated 24****h post incubation.

### Sediment particle size analysis

Sediment grain size was determined by laser diffraction after 1****min sonication to separate particles, using a Malvern Hydro 2000 MU particle size analyser in conjunction with the Mastersizer 2000 software. Three replicate sediment samples from each site were pooled and homogenised. Approximately 1****g of sediment was added to the particle size analyser and 3 independent size determinations were made. This was repeated 3 times using the same pooled sample to determine an overall average.

### Determination of sediment organic matter content

The loss on ignition method (LOI) was used to determine organic matter content of sediment samples. Three replicate sediment samples from each site were pooled and homogenised. Approximately 20 g of fresh sediment from each sample was placed in a pre-weighed crucible and dried at 95°C for 24****h. Approximately 4****g of the resultant dried sediment was pre-weighed, transferred to another crucible and placed into a muffle furnace at 550°C for 4****h. Organic matter content was calculated as the difference between the weight of the dry sediment and weight of the residue post-combustion. This was repeated 3 times using the same pooled sample to determine an overall average. Moisture content per g of fresh sediment was determined by calculating the percentage difference between wet weight and dry weight after 24****h at 95°C (Table S2).

### *In situ* physico-chemical measurements of estuarine transect sample sites

Conductivity (calculated to practical salinity units (PSU)), temperature and depth measurements were recorded *in* **** *situ* using a YSI 600LS CTD scanner attached to a YSI 650 MDS data logger. This was deployed at each sample site in parallel with collection of water and sediment samples. Salinity and temperature measurements were recorded from the water immediately above the sediment bed and 0.2****m below the water surface. (Conductivity readings were calculated to determine salinity) (Table S3).

### DNA extraction, 16S rRNA gene PCR and sequencing of bacterial isolates

To validate the identity of bacterial colonies on selective microbiological medium, DNA was extracted from a total of 30 isolates that matched or were similar to the expected colony morphology/phenotype for *E. coli*, enterococci, *Campylobacter* and *Vibrio spp*. for 16S rRNA gene PCR amplification and sequencing. For *E. coli* and enterococci, little variation in colony morphology and phenotype was observed across all of the colonies counted, and so only a small number of isolates were sequenced for confirmation.

DNA was extracted from 30 isolated bacterial colonies using the ISOLATE genomic DNA mini kit (Bioline Reagents Ltd., London, UK) following the manufacturer’s protocol. Agarose gel electrophoresis (1%) was used to visualise the DNA extracted from each bacterial isolate. Subsequently, the 16S rRNA gene of each isolate was amplified via PCR using the primer pair pA (5′-AGAGTTTGATCCTGGCTCAG-3′) and pH (5′-AAGGAGGTGATCCAGCCGCA-3′) (45). PCR reactions consisted of 10 pmol of each primer (pA and pH), 1x MyTaq red mix (Bioline), approximately 100****ng of template DNA and ddH_2_O to a total reaction volume of 50 µl. The PCR conditions were as follows: an initial denaturation step at 95°C for 1****min, followed by 35 cycles of 95°C for 15****s, 55°C for 15 s, 72°C for 10****s and a final hold at 4°C. PCR amplicons were visualised using 1% agarose gel. The expected 16S rRNA gene amplicon size was approximately 1500 bp and amplicons of the expected size were subsequently excised from the agarose gel and purified using the Isolate PCR and Gel Kit (Bioline Reagents Ltd., London, UK) following the manufacturer’s instructions. Purified 16S rRNA gene amplification products for each bacterial isolate were sequenced in both the forward and reverse orientation by Macrogen Europe (Netherlands). The Geneious Pro software package, Geneious 6.1 version created by Biomatters, (Available from http://www.geneious.com/) was used to quality clip each sequence and assemble the forward and reverse reads of each strain into a contiguous sequence. The sequence identity of each contig was determined using NCBI BLASTn. Identification of sequenced strains is given in Table S4 of the supplemental material.

### Statistical analysis

Using the Statistical Package for Social Sciences SPSS v20, (IBM Corp., Armonk. NY), basic correlations were performed using the average data calculated for each site to determine the relationships between cultured bacterial abundance in sediments and water with different tested parameters. The non-parametric Spearman Rank Correlation Coefficient (r_s_) was used due to the data being not normally distributed.

## Results

### The relationship between PIB abundance, sediment grain size and organic content

**Figure 2:**
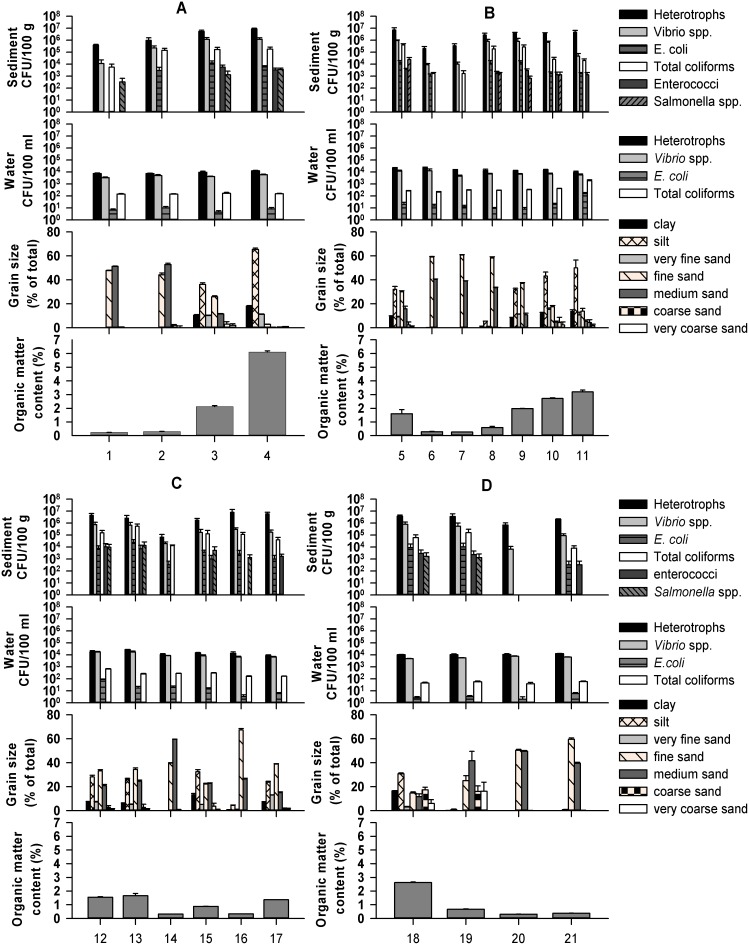
Bacterial abundance (CFU/100 g wet weight) compared to sediment grain size, organic matter content in sediments and bacterial abundance in the water column (CFU/100 ml), across four transverse transects. (A) Transect 1, (B) Transect 2, (C) Transect 3, (D) Transect 4. The X-axis represents sample points (n = 3 replicate samples for A, B, C and D except for sediment samples site 13, n = 2), mean values are plotted and error bars represent the SEM).

There were marked spatial differences in the abundance of sediment-associated PIB across all of the 21 sample sites within the estuary as determined by culture counts on selective medium (Table 1). Mean densities of *E. coli* and total coliforms in sediments ranged from 0 to 2.4×10^4^ CFU/100 g and 0 to 5.4×10^5^ CFU/100 g wet weight, respectively (for all mean bacterial densities from all sampling sites see Table S5 in the supplemental material). Enterococci and *Salmonella* abundance varied from 0 to 1×10^4^ CFU/100 g and 0 to 2.5×10^4^ CFU/100 g wet weight, respectively. *Vibrio* spp. were detected at all 21 sample sites (6.7×10^3^ to 1.2×10^6^ CFU/100****g wet weight) and this reflects their status as indigenous members of marine and aquatic environments. In addition, direct colony counts on *Campylobacte*r selective media indicated the presence of 0 to 3.5×10^4^ CFU/100 g wet weight. However, 16S rRNA gene sequencing of putative *Campylobacter* spp. isolated from a subsequent sampling survey on the same selective medium revealed that none of the sequenced isolates were *Campylobacter* spp. (Table S4). Culturable heterotrophic bacteria were enumerated as a proxy for the abundance of the indigenous estuarine microbial community and their abundance ranged from 6.6×10^4^ to 8.7×10^6^ CFU/100 g wet weight. The enumeration of culturable heterotrophic bacteria alongside our target PIB groups enabled analysis of the relationship between total culturable heterotrophic bacteria counts and PIB counts in sediments, these data demonstrate that the abundance of heterotrophic bacteria within the sediments showed significant positive correlations with the abundance of all PIB groups measured (Table S6). Sediment grain size composition within sediment samples ranged from clay (0 to 18%), silt (0 to 65%), very fine sand (0 to 15%), fine sand (3 to 67%) medium sand (0 to 59%), coarse sand (0 to 17%) and very coarse sand (0 to 16%). The organic matter content of the sediment samples varied from 0.3 to 6% across the sample sites (Figure 2).

**Table 1:** Average abundance of culturable pathogen indicator bacteria in sediment (CFU/100 g wet weight) and water (CFU/100****ml) across 21 estuarine sample sites.

Bacterial group	Sediment	Water	Fold - difference
*E. coli*	5.9×10^3^	2.1×10^1^	281
Total coliforms	1.3 x10^5^	3.0×10^2^	433
*Vibrio* spp.	4.5×10^5^	7.8×10^3^	58

The abundance of each cultured bacterial group within the sediments showed significant positive correlations with the abundance of all other measured bacterial groups (Table S6). *Vibrio* spp. were also more abundant in sediments that had higher densities of FIB. In addition, the abundance of all isolated PIB groups showed a significant positive correlation with both sediment clay content (grain size <4 µm) (*E. coli*, enterococci, total coliforms, and *Vibrio* spp. r_s_ = 0.543 (p<0.011) r_s_ = 0.664 (p<0.001), r_s_ = 0.495 (p<0.023), r_s_ = 0.663 (p<0.001) respectively) and silt content (grain size 4 µm–63 µm) (*E. coli*, enterococci, total coliforms and *Vibrio* spp., r_s_ = 0.570 (p<0.007) r_s_ = 0.687 (p<0.001), r_s_ = 0.547 (p<0.010), r_s_ = 0.688 (p<0.001) respectively). Significant negative correlations were observed between the abundance of all PIB groups with fine sand (125 µm–250 µm) and medium sand (250 µm–500 µm) (Table S7). Sediments with high amounts of clay and silt along with very fine sand contained the greatest proportion of organic material (significant positive correlation; organic matter content and clay, r_s_ = 0.917 (p<0.001), organic matter content and silt, r_s_ = 0.926 (p<0.001), organic matter content and very fine sand, r_s_ = 0.810 (p<0.001). Significant negative correlations were evident between organic matter content and fine sand and also organic matter content and medium sand (Table S8). Sediments with high organic matter therefore also showed significant positive correlations with the abundance of all isolated PIB (Table S9).

### Comparison of PIB abundance between sediment and water samples

The average abundance data for culturable PIB (*E. coli*, total coliforms and *Vibrio* spp.) in sediment and water samples across all 21 sample sites revealed that *E. coli*, coliforms and *Vibrio* spp. were 281, 433 and 58-fold more abundant in the sediment (CFU/100 g) than the water column (CFU/100 ml), respectively (Table 1).

### The effect of depth, salinity and temperature on PIB abundance

Collection of water samples was initiated at slack water, with a range of water column depths from 0.5 to 12.3 m across all sites. Salinity and temperature measurements taken from above the bottom sediment varied between 9.3 to 30.0 psu and 10.8 to 11.2°C, respectively. At 0.2 m below the water surface the temperature varied from 10.8 to 11.2°C and salinity ranged from 10.1 to 27.8 psu. Bacterial abundance within the sediments revealed no significant correlations with any of the physico-chemical parameters measured (Table S10). *Vibrio* spp. enumerated from the water samples were the only cultured species to show significant correlations with depth, temperature or salinity. Temperature had a significant negative correlation with the abundance of *Vibrio* spp. (r_s_ = 0.614 p<0.003), while salinity had a significant positive correlation with abundance (r_s_ = 0.544 p<0.011) (Table S11).

### Taxonomic identification of bacterial isolates using 16S rRNA gene sequencing

None of the isolates derived from the selective medium for *Campylobacter* (n = 7) were positively identified as the target group. However, 91% of *E. coli* (n = 11), 100% of enterococci (n = 3) and 67% of *Vibrio* (n = 9) isolates were positively identified as the target group (Table S4). No sequence data were obtained for *Salmonella* isolates and they should therefore be considered as presumptive *Salmonella* spp.

## Discussion

Results from this study demonstrate that sediments represent a significant reservoir for PIB and that sediment characteristics (grain size and organic matter content) influence spatial variations in PIB abundance in estuarine environments. Here, clay and silt fractions comprised a higher organic matter content in comparison to medium and coarse sand and harboured significantly higher densities of PIB. Previous studies have also concluded that the presence of finer sediment particles can have a positive impact on FIB abundance [25], [46] and that organic material is a contributing factor to the prolonged persistence and survival of FIB in sediments [24], [47], [48]. However, almost all of the previous studies focus specifically on ‘classic’ faecal indicators such as *E. coli*, coliforms and enterococci, and here we have expanded knowledge on comparative PIB–sediment interactions with sediments for a greater repertoire of bacterial groups.

Results revealed there was not only a large spatial variation in PIB abundance that correlated to sediment grain size and organic matter content, but PIB abundance was also found to be significantly higher in the sediments when compared to the overlying water column, with levels of *E. coli* reaching over 3 orders of magnitude higher in the sediment. It is well documented that bacterial association with sediments circumvents the negative effects of environmental stresses, such as providing protection from UV light (34), which in turn can augment the survival and even growth of FIB. For example, DNA fingerprinting analyses performed on *E. coli* populations in beach sand and sediment revealed the possibility that some strains may have become naturalized to this environment [52] and growing evidence also suggests that there may be free-living strains of FIB surviving and multiplying within the water column of environmental waters, independently of a host [53].

The abundance of *E. coli* and enterococci in sediments had a significant positive correlation with the abundance of other PIB. Consequently, the enumeration of FIB as the ‘classic’ bacterial indicators currently conducted as part of routine water quality monitoring represent suitable indicators for the presence of other PIB groups. *E. coli* and enterococci are the predominant indicator species for monitoring faecal pollution of aquatic environments within the European Union (EU) despite numerous studies highlighting the differential environmental survival of different FIB groups [48], [51], in addition to different strains of the same group [54]. In this study, the presence and abundance of enterococci had a stronger significant positive correlation with silt and clay when compared with other FIB, which may suggest greater survival times of enterococci under favourable conditions that certain sediment types may provide. However, it is well established that the survival times of different species, and even strains of the same species, varies considerably in aquatic environments, both between and within species, and this must be taken into consideration. For example, in comparison to other FIB, enterococci survive for longer periods in sediments [48] and within estuarine environments [31], and it has been proposed that the increase in survival under harsh conditions may be due to their membrane composition [55], [56]. Such evidence suggests that enterococci may be a more robust indicator of the persistence of faecal contamination rather than more recent contamination within coastal and estuarine environments.

The enumeration of total heterotrophs revealed significant positive correlations with all other cultured PIB groups and therefore the same trends were seen within the indigenous microbial community (Table S6). It should be noted that despite obtaining high colony counts on selective microbiological media for *Campylobacter* spp., 16S rRNA gene sequence analysis of some of these isolates suggested that none were *Campylobacter* spp. These data highlight the potential pitfalls of culture-based analyses of microbial taxa using a selective microbiological medium, and care must be taken when interpreting microbial culture counts. Despite this, the sequenced isolates of enterococci, *Vibrio* spp. and *E. coli* indicate that these media were selective for the desired bacterial target groups (Table S4). Little variation in temperature and salinity could be explained by the small geographical variation along each transect. There was no significant correlation between temperature and salinity and the abundance of PIB, with the exception of *Vibrio* spp. enumerated from the water column. Here, *Vibrio* spp. had a significant positive correlation with salinity and a significant negative correlation with temperature, supporting previous proposals that temperature and salinity have important implications for *Vibrio* spp. population dynamics [30]. The depth at which sediment samples were taken had no impact on the abundance of PIB enumerated from the sediments.

This study highlights the risk of periodic elevated FIB and other PIB in the water column due to the resuspension of microbial contaminated sediments. Furthermore, the risk of microbial pollution from the resuspension of sediments is not taken into consideration when assessing microbial pollution of recreational and shellfish harvesting waters. The time and place of sampling in addition to tidal and hydrodynamic conditions may impact upon FIB concentrations in the water column. Despite several reports on the importance of sediments as a reservoir for FIB [28], [29], the enumeration of waterborne FIB has received much more attention. Quilliam *et al*., (2011) revealed significant spatial variations in waterborne *E. coli* numbers on contrasting sides of the same four transverse transects of the Conwy estuary studied here, indicating that significantly different levels of microbial pollution were present in the east and west sides of the river, and the localised re-suspension of sediment associated FIB into the water column was one proposed explanation for such contrasting FIB counts in the water column [45]. Despite this, the dynamics of sediment transport and re-suspension in relation to PIB concentrations is poorly understood. The attachment of PIB to particulate matter in the water column provides a platform for transportation and downward flux to the bed sediment, cyclical changes in tidal flow and the salinity of the water will also impact the downward flow of particle-associated PIB to the bed sediments. Conversely, under certain hydrodynamic conditions (e.g. tidal cycles and storm flow events), bed stress and turbulence can also impart the re-suspension of sediments and subsequent transportation and deposition of particle associated FIB to other areas of the estuary. Due to the hydrodynamics of an estuarine system, fine sediments are usually deposited around the banks of the basin [49] and our data support this trend, with finer particles detected in greater abundance in sediments on the east and west sides of the estuary and coarser sand deposited in the central channel. FIB levels in water can be affected by other factors such as temperature [33], exposure to sunlight and salinity [31], nutrient concentrations [5], predation by protozoan [50] and competition [51]. However, here we demonstrate that sediments also contribute to the distribution of FIB and other PIB in an estuarine environment.

Experimental results confirm that estuarine sediments harbour PIB, which may potentiate their prolonged persistence and survival in this environment. This study also identifies areas of high microbial contamination within the Conwy estuary, which highlights the risk of sediment and PIB resuspension back into the water column under turbulent hydrodynamic conditions that result in sediment bed stress and promote the erosion of sediments. To our knowledge, this is the first comprehensive study of the co-occurrence of *E. coli*, coliforms, enterococci, *Salmonella* and *Vibrio* spp. (PIB) in relation to sediment composition. These data show that all PIB groups studied are strongly correlated with the presence of clay, silt and organic matter content in sediments. In addition, the presence of *E. coli* and total coliforms strongly correlates with the abundance of the other PIB tested, both allochthonous and autochthonous, suggesting that culture-based determinations of *E. coli* and coliform abundance in sediments represent a useful surrogate for the presence of other PIB groups.

## Conclusion

Faecal contamination in aquatic environments is currently assessed by measuring culturable *E. coli* and enterococci counts in water samples only. Here, we demonstrate that sediment composition, specifically clay, silt and organic matter content, are linked with greater PIB abundance in sediments. The enhanced abundance of viable PIB in sediments therefore has implications for water quality and public health when considering the potential for resuspension of bed sediments, particularly finer particles such as clay and silt that we have demonstrated to contain higher concentrations of PIB. It may therefore be necessary to incorporate PIB loadings in bottom sediments into routine monitoring protocols and hydrodynamic models to adequately assess their risk to human health. The detection of spatial variations of PIB within sediments also highlights the necessity for further research on the interactions of pathogens with sediments and their role in the survival, persistence and transportation of PIB within environmental waters.

## Supporting Information

Table S1 **Selective media used to enumerate target bacterial groups.**
(DOCX)

Table S2 **Sediment dry weight determined from 1 g^−1^ wet weight.**
(DOCX)

Table S3 **Details the depth at which the sediment samples were collected, salinity measurements taken for both water samples (0.2 m from the surface) and directly above the sediment samples, calculated as Practical Salinity Units (PSU).** Temperature was recorded at a depth of 0.2 m from the surface and directly above the sediment.
(DOCX)

Table S4 **Identification of sequenced isolates.**
(DOCX)

Table S5 **Bacteria counts, sediment (CFU/100 g) versus water (CFU/100 ml).** Data shown as mean (n = 3, sample point 13 n = 2 for sediment).
(DOCX)

Table S6 **Correlation coefficient (r_s_) matrix demonstrating the relationship between the abundance of each cultured bacterial group within estuarine sediments (n = 21).**
(DOCX)

Table S7 **Correlation coefficient (r_s_) matrix demonstrating the relationship between the abundance of each cultured bacterial group within estuarine sediments and sediment grain size (n = 21).**
(DOCX)

Table S8 **Correlation coefficient (r_s_) matrix demonstrating the relationship between estuarine sediment grain size (%) and organic matter content (%) (n = 21).**
(DOCX)

Table S9 **Correlation coefficient (r_s_) matrix demonstrating the relationship between the abundance of each cultured bacterial group within estuarine sediments and sediment organic matter content (n = 21).**
(DOCX)

Table S10 **Correlation coefficient (r_s_) matrix demonstrating the relationship between the abundance of each cultured bacterial group within estuarine sediments and physico-chemical parameters measured directly above the bottom sediments (n = 2).**
(DOCX)

Table S11 **Correlation coefficient (r_s_) matrix demonstrating the relationship between the abundance of each cultured bacterial group within estuarine water and physico-chemical parameters measured at 0.2 m depth (n = 21).**
(DOCX)
