# Supplementary material for: Sediment Composition Influences Spatial Variation in the Abundance of Human Pathogen Indicator Bacteria within an Estuarine Environment
Source: PLoS One. 2014 Nov 14;9(11):e112951. doi: 10.1371/journal.pone.0112951 (PMC4232572; doi:10.1371/journal.pone.0112951)
Supplement: Table S1 — Selective media used to enumerate target bacterial groups. (DOCX) [file pone.0112951.s001.docx]

**Table S1.** Selective media used to enumerate target bacterial groups.

| Bacteria group | Media | Incubation period |
| --- | --- | --- |
| *E.coli* / coliforms* | Harlequin (LabM HAL008) | 24 h 37 °C |
| *Salmonella* spp. | Harlequin Salmonella ABC Medium (LabM HAL001) | 24 h 37 °C |
| *Enterococcus spp.* | Slanetz & Bartley Medium (LabM LAB166) | 48 h 37 °C |
| *Campylobacter* spp. | Campylobacter Blood-free Selective Medium (LabM LAB112) supplemented with cefoperazone and amphotericin (LabM X112) | 48 h 37 °C under microaerophilic conditions using Campygen sachets (Oxoid, CN0035) |
| Total heterotrophs | Marine Agar (Deben Diagnostics Ltd, Ipswich UK) | 24 h Room temperature |
| *Vibrio* spp. | Cholera Medium TCBS (Oxoid Ltd, Basingstoke, UK) | 24 h Room temperature |
