# Supplementary material for: Sediment Composition Influences Spatial Variation in the Abundance of Human Pathogen Indicator Bacteria within an Estuarine Environment
Source: PLoS One. 2014 Nov 14;9(11):e112951. doi: 10.1371/journal.pone.0112951 (PMC4232572; doi:10.1371/journal.pone.0112951)
Supplement: Table S2 — Sediment dry weight determined from 1 g−1 wet weight. (DOCX) [file pone.0112951.s002.docx]

**Table S2.** Sediment dry weight determined from 1 g^-1^ wet weight.

| Sample site | Sediment  dry weight  (1 g^-1^) |
| --- | --- |
| 1 | 0.950127 |
| 2 | 0.949253 |
| 3 | 0.912751 |
| 4 | 0.844045 |
| 5 | 0.920252 |
| 6 | 0.94133 |
| 7 | 0.945941 |
| 8 | 0.940672 |
| 9 | 0.919224 |
| 10 | 0.914467 |
| 11 | 0.891909 |
| 12 | 0.900297 |
| 13 | 0.91571 |
| 14 | 0.934392 |
| 15 | 0.946164 |
| 16 | 0.941073 |
| 17 | 0.935374 |
| 18 | 0.909405 |
| 19 | 0.943176 |
| 20 | 0.947442 |
| 21 | 0.947442 |
