# Supplementary material for: Sediment Composition Influences Spatial Variation in the Abundance of Human Pathogen Indicator Bacteria within an Estuarine Environment
Source: PLoS One. 2014 Nov 14;9(11):e112951. doi: 10.1371/journal.pone.0112951 (PMC4232572; doi:10.1371/journal.pone.0112951)
Supplement: Table S3 — Details the depth at which the sediment samples were collected, salinity measurements taken for both water samples (0.2 m from the surface) and directly above the sediment samples, calculated as Practical Salinity Units (PSU). Temperature was recorded at a depth of 0.2 m from the surface and directly above the sediment. (DOCX) [file pone.0112951.s003.docx]

**Table S3.** Details the depth at which the sediment samples were collected, salinity measurements taken for both water samples (0.2 m from the surface) and directly above the sediment samples, calculated as Practical Salinity Units (PSU). Temperature was recorded at a depth of 0.2 m from the surface and directly above the sediment.

|  | Depth (m) | Salinity (water)  PSU | Salinity (sediment ) PSU | Temperature (water)  (°C) | Temperature (sediment)  (°C) |
| --- | --- | --- | --- | --- | --- |
| 1 | 5.005 | 18.0327 | 25.8697 | 11.2235 | 11.023 |
| 2 | 3.566 | 17.9367 | 25.7881 | 11.1794 | 11.0094 |
| 3 | 5.191 | 20.0538 | 25.8161 | 11.2457 | 11.023 |
| 4 | 1.228 | 19.8106 | 23.1885 | 11.1724 | 11.1887 |
| 5 | 4.864 | 23.7029 | 25.0603 | 11.1462 | 11.1034 |
| 6 | 3.972 | 23.7128 | 26.4271 | 11.1238 | 10.9669 |
| 7 | 2.083 | 19.5844 | 26.7968 | 11.2352 | 10.9224 |
| 8 | 3.981 | 21.4085 | 27.6613 | 11.1032 | 10.8209 |
| 9 | 4.14 | 22.5498 | 27.4436 | 11.0665 | 10.8411 |
| 10 | 0.505 | 22.3528 | 23.4288 | 11.0709 | 11.1272 |
| 11 | 0.461 | 10.0841 | 9.3445 | 10.9196 | 10.9199 |
| 12 | 4.361 | 25.3284 | 27.0885 | 10.8457 | 10.9056 |
| 13 | 12.321 | 26.144 | 27.8303 | 10.8312 | 10.8992 |
| 14 | 6.295 | 23.7251 | 27.9589 | 10.9632 | 10.9028 |
| 15 | 3.981 | 24.2388 | 26.2542 | 10.9852 | 10.9684 |
| 16 | 4.802 | 24.8404 | 26.8336 | 10.9784 | 10.9171 |
| 17 | 1.431 | 23.4513 | 26.1675 | 11.0741 | 10.963 |
| 18 | 10.196 | 24.2663 | 29.9778 | 11.1424 | 10.7806 |
| 19 | 4.44 | 26.0472 | 29.6304 | 10.9383 | 10.7808 |
| 20 | 4.687 | 27.7847 | 29.7162 | 10.7859 | 10.8518 |
| 21 | 4.643 | 26.2878 | 29.273 | 11.1253 | 10.8866 |
