# Supplementary material for: Sediment Composition Influences Spatial Variation in the Abundance of Human Pathogen Indicator Bacteria within an Estuarine Environment
Source: PLoS One. 2014 Nov 14;9(11):e112951. doi: 10.1371/journal.pone.0112951 (PMC4232572; doi:10.1371/journal.pone.0112951)
Supplement: Table S4 — Identification of sequenced isolates. (DOCX) [file pone.0112951.s004.docx]

**Table S4.** Identification of sequenced isolates.

| Presumed | Closest blast search identity match | Querycov/  Max ident % | Sequence length |
| --- | --- | --- | --- |
| *Campylobacter* spp. | *Acinetobacter calcoaceticus* | 99 / 100 | 1425 |
| *Campylobacter* spp. | *Acinetobacter calcoaceticus* | 100 / 100 | 1409 |
| *Campylobacter* spp. | *Acrobacter butzleri* | 100 / 99 | 1250 |
| *Campylobacter* spp. | *Ochrobactrum intermedium* | 99 / 100 | 1382 |
| *Campylobacter* spp. | *Escherichia coli* | 100 / 99 | 1440 |
| *Campylobacter* spp. | *Agrobacterium tumefaciens* | 100 / 100 | 1372 |
| *Campylobacter* spp. | *Pseudomonas flourescens* | 100 / 99 | 1416 |
| *Escherichia coli* | *Escherichia coli* | 100 / 100 | 1425 |
| *Escherichia coli* | *Escherichia coli* | 100 / 99 | 1420 |
| *Escherichia coli* | *Escherichia coli* | 100 / 99 | 1430 |
| *Escherichia coli* | *Escherichia coli* | 100 / 99 | 1428 |
| *Escherichia coli* | *Escherichia coli* | 100 / 99 | 1428 |
| *Escherichia coli* | *Escherichia coli* | 100 / 99 | 1428 |
| *Escherichia coli* | *Escherichia coli* | 100 / 99 | 1437 |
| *Escherichia coli* | *Escherichia coli* | 100 / 99 | 1441 |
| *Escherichia coli* | *Escherichia coli* | 100 / 99 | 1438 |
| *Escherichia coli* | *Escherichia coli* | 100 / 99 | 1428 |
| *Escherichia coli* | *Acinetobacter spp.* | 100 / 99 | 1434 |
| *Vibrio* spp. | *Shewanella* spp. | 100 / 99 | 1430 |
| *Vibrio* spp. | *Vibrio splendidus* | 99 / 99 | 1448 |
| *Vibrio* spp. | *Vibrio splendidus* | 100 / 99 | 1450 |
| *Vibrio* spp. | *Vibrio splendidus* | 100 / 99 | 1440 |
| *Vibrio* spp. | *Vibrio splendidus* | 100 / 99 | 1451 |
| *Vibrio* spp. | *Shewanella piezotolerans* | 100 / 99 | 1439 |
| *Vibrio* spp. | *Vibrio artabroborum* | 100 / 97 | 1449 |
| *Vibrio* spp. | *Photobacterium frigidiphilum* | 100 / 98 | 1442 |
| *Vibrio* spp. | *Vibrio* spp. | 100 / 96 | 1445 |
| *Enterococcus* spp. | *Enterococcus faecalis* | 100 / 99 | 1358 |
| *Enterococcus* spp. | *Enterococcus faecalis* | 100 / 99 | 1447 |
| *Enterococcus* spp. | *Enterococcus hirae* | 100 / 99 | 1335 |
