# Supplementary material for: Sediment Composition Influences Spatial Variation in the Abundance of Human Pathogen Indicator Bacteria within an Estuarine Environment
Source: PLoS One. 2014 Nov 14;9(11):e112951. doi: 10.1371/journal.pone.0112951 (PMC4232572; doi:10.1371/journal.pone.0112951)
Supplement: Table S5 — Bacteria counts, sediment (CFU/100 g) versus water (CFU/100 ml). Data shown as mean (n = 3, sample point 13 n = 2 for sediment). (DOCX) [file pone.0112951.s005.docx]

**Table S5.** Bacteria counts, sediment (CFU/100 g) versus water (CFU/100 ml). Data shown as mean (n=3, sample point 13 n=2 for sediment).

| Sample Site | 1 | 2 | 3 | 4 | 5 | 6 | 7 | 8 | 9 | 10 | 11 | 12 | 13 | 14 | 15 | 16 | 17 | 18 | 19 | 20 | 21 |
| --- | --- | --- | --- | --- | --- | --- | --- | --- | --- | --- | --- | --- | --- | --- | --- | --- | --- | --- | --- | --- | --- |
| *E. coli*  CFU/100 g | 0 | 3000 | 12667 | 6000 | 13667 | 1000 | 0 | 12000 | 13333 | 1667 | 1667 | 7667 | 24000 | 333 | 3667 | 2667 | 1000 | 9000 | 10667 | 0 | 333 |
| *E.coli* CFU/100 ml | 7 | 10 | 5 | 9 | 20 | 15 | 13 | 9 | 8 | 21 | 159 | 88 | 17 | 19 | 14 | 3 | 6 | 3 | 3 | 2 | 6 |
| Coliforms CFU/100 g | 5667 | 145667 | 173667 | 185667 | 399333 | 1667 | 1667 | 191000 | 281667 | 26000 | 20667 | 153667 | 546000 | 13000 | 126000 | 114667 | 39333 | 60333 | 156000 | 0 | 8000 |
| Coliforms CFU/100 ml | 147 | 147 | 171 | 157 | 265 | 215 | 311 | 297 | 333 | 423 | 1865 | 664 | 255 | 281 | 302 | 161 | 163 | 45 | 57 | 39 | 59 |
| *Vibrio* spp.  CFU/100 g | 11667 | 224167 | 1208333 | 1204167 | 934167 | 9167 | 10000 | 801667 | 817500 | 676667 | 48333 | 774167 | 671250 | 19167 | 164167 | 300833 | 173333 | 795833 | 546667 | 6667 | 87500 |
| *Vibrio* spp.  CFU/100 ml | 3467 | 5067 | 4200 | 6067 | 12200 | 12933 | 4667 | 7000 | 6733 | 7000 | 5667 | 17267 | 17200 | 8600 | 8133 | 6733 | 6733 | 4867 | 5733 | 7600 | 6467 |

| Presumed | Closest blast search identity match | Querycov/  Max ident % | Sequence length |
| --- | --- | --- | --- |
| *Campylobacter* spp. | *Acinetobacter calcoaceticus* | 99 / 100 | 1425 |
| *Campylobacter* spp. | *Acinetobacter calcoaceticus* | 100 / 100 | 1409 |
| *Campylobacter* spp. | *Acrobacter butzleri* | 100 / 99 | 1250 |
| *Campylobacter* spp. | *Ochrobactrum intermedium* | 99 / 100 | 1382 |
| *Campylobacter* spp. | *Escherichia coli* | 100 / 99 | 1440 |
| *Campylobacter* spp. | *Agrobacterium tumefaciens* | 100 / 100 | 1372 |
| *Campylobacter* spp. | *Pseudomonas flourescens* | 100 / 99 | 1416 |
| *Escherichia coli* | *Escherichia coli* | 100 / 100 | 1425 |
| *Escherichia coli* | *Escherichia coli* | 100 / 99 | 1420 |
| *Escherichia coli* | *Escherichia coli* | 100 / 99 | 1430 |
| *Escherichia coli* | *Escherichia coli* | 100 / 99 | 1428 |
| *Escherichia coli* | *Escherichia coli* | 100 / 99 | 1428 |
| *Escherichia coli* | *Escherichia coli* | 100 / 99 | 1428 |
| *Escherichia coli* | *Escherichia coli* | 100 / 99 | 1437 |
| *Escherichia coli* | *Escherichia coli* | 100 / 99 | 1441 |
| *Escherichia coli* | *Escherichia coli* | 100 / 99 | 1438 |
| *Escherichia coli* | *Escherichia coli* | 100 / 99 | 1428 |
| *Escherichia coli* | *Acinetobacter spp.* | 100 / 99 | 1434 |
| *Vibrio* spp. | *Shewanella* spp. | 100 / 99 | 1430 |
| *Vibrio* spp. | *Vibrio splendidus* | 99 / 99 | 1448 |
| *Vibrio* spp. | *Vibrio splendidus* | 100 / 99 | 1450 |
| *Vibrio* spp. | *Vibrio splendidus* | 100 / 99 | 1440 |
| *Vibrio* spp. | *Vibrio splendidus* | 100 / 99 | 1451 |
| *Vibrio* spp. | *Shewanella piezotolerans* | 100 / 99 | 1439 |
| *Vibrio* spp. | *Vibrio artabroborum* | 100 / 97 | 1449 |
| *Vibrio* spp. | *Photobacterium frigidiphilum* | 100 / 98 | 1442 |
| *Vibrio* spp. | *Vibrio* spp. | 100 / 96 | 1445 |
| *Enterococcus* spp. | *Enterococcus faecalis* | 100 / 99 | 1358 |
| *Enterococcus* spp. | *Enterococcus faecalis* | 100 / 99 | 1447 |
| *Enterococcus* spp. | *Enterococcus hirae* | 100 / 99 | 1335 |

**Table S2** Identification of sequenced isolates.

|  | *E. coli* | Total coliforms | *Salmonella* spp. | Enterococci | Heterotrophs | *Vibrio* spp. | |
| --- | --- | --- | --- | --- | --- | --- | --- |
| *E. coli* | 1.000 |  |  |  |  |  | |
| Total coliforms | 0.945^**^ | 1.000 |  |  |  |  | |
| *Salmonella* spp. | 0.763^**^ | 0.759^**^ | 1.000 |  |  |  | |
| Enterococci | 0.817^**^ | 0.780^**^ | 0.729^**^ | 1.000 |  |  | |
| Heterotrophs | 0.536^*^ | 0.574^**^ | 0.476^*^ | 0.645^**^ | 1.000 |  | |
| *Vibrio* spp. | 0.847^**^ | 0.859^**^ | 0.709^**^ | 0.817^**^ | 0.720^**^ | 1.000 | |
| **. Correlation is significant at the 0.01 level (2-tailed). | | | | | | |  |
| *. Correlation is significant at the 0.05 level (2-tailed). | | | | | | |  |

**Table S3** Correlation coefficient (r_s_) matrix demonstrating the relationship between the abundance of each cultured bacterial group within estuarine sediments (n = 21).

|  | *E. coli* | Total coliforms | *Salmonella* spp. | Enterococci | *Vibrio* spp. | Clay  (<4 µm) | Silt  (4 µm-63 µm) | Very fine sand (63 µm-125 µm) | Fine sand  (125 µm-250 µm) | Medium sand  (250 µm-500 µm) | Coarse sand  (500 µm-1000 µm) | Very coarse Sand  (1000 µm-2000 µm) |
| --- | --- | --- | --- | --- | --- | --- | --- | --- | --- | --- | --- | --- |
| *E. coli* | 1.000 |  |  |  |  |  |  |  |  |  |  |  |
| Total coliforms | 0.945^**^ | 1.000 |  |  |  |  |  |  |  |  |  |  |
| *Salmonella* spp. | 0.763^**^ | 0.759^**^ | 1.000 |  |  |  |  |  |  |  |  |  |
| Enterococci | 0.817^**^ | 0.780^**^ | 0.729^**^ | 1.000 |  |  |  |  |  |  |  |  |
| *Vibrio* spp. | 0.847^**^ | 0.859^**^ | 0.709^**^ | 0.817^**^ | 1.000 |  |  |  |  |  |  |  |
| Clay | 0.543^*^ | 0.495^*^ | 0.568^**^ | 0.664^**^ | 0.663^**^ | 1.000 |  |  |  |  |  |  |
| Silt | 0.570^**^ | 0.547^*^ | 0.578^**^ | 0.687^**^ | 0.688^**^ | 0.958^**^ | 1.000 |  |  |  |  |  |
| Very fine sand | 0.432 | 0.446^*^ | 0.368 | 0.625^**^ | 0.605^**^ | 0.804^**^ | 0.828^**^ | 1.000 |  |  |  |  |
| Fine sand | -0.457^*^ | -0.403 | -0.462^*^ | -0.618^**^ | -0.504^*^ | -0.795^**^ | -0.840^**^ | -0.539^*^ | 1.000 |  |  |  |
| Medium sand | -0.450^*^ | -0.416 | -0.419 | -0.650^**^ | -0.648^**^ | -0.899^**^ | -0.923^**^ | -0.935^**^ | 0.668^**^ | 1.000 |  |  |
| Coarse sand | 0.334 | 0.223 | 0.361 | 0.448^*^ | 0.266 | 0.486^*^ | 0.517^*^ | 0.214 | -0.774^**^ | -0.292 | 1.000 |  |
| Very coarse sand | 0.443^*^ | 0.353 | 0.398 | 0.634^**^ | 0.504^*^ | 0.625^**^ | 0.696^**^ | 0.461^*^ | -0.851^**^ | -0.578^**^ | 0.864^**^ | 1.000 |
| **. Correlation is significant at the 0.01 level (2-tailed). | | | | | | | | | | | | |
| *. Correlation is significant at the 0.05 level (2-tailed). | | | | | | | | | | | | |

**Table S4** Correlation coefficient (r_s_) matrix demonstrating the relationship between the abundance of each cultured bacterial group within estuarine sediments and sediment grain size (n=21).

|  | Organic matter | Clay | Silt | Very fine sand | Fine sand | Medium sand | Coarse sand | Very coarse sand |
| --- | --- | --- | --- | --- | --- | --- | --- | --- |
| Organic matter | 1.000 |  |  |  |  |  |  |  |
| Clay | 0.917^**^ | 1.000 |  |  |  |  |  |  |
| Silt | 0.926^**^ | 0.958^**^ | 1.000 |  |  |  |  |  |
| Very fine sand | 0.810^**^ | 0.804^**^ | 0.828^**^ | 1.000 |  |  |  |  |
| Fine sand | -0.834^**^ | -0.795^**^ | -0.840^**^ | -0.539^*^ | 1.000 |  |  |  |
| Medium sand | -0.903^**^ | -0.899^**^ | -0.923^**^ | -0.935^**^ | 0.668^**^ | 1.000 |  |  |
| Coarse sand | 0.527^*^ | 0.486^*^ | 0.517^*^ | 0.214 | -0.774^**^ | -0.292 | 1.000 |  |
| Very coarse sand | 0.736^**^ | 0.625^**^ | 0.696^**^ | 0.461^*^ | -0.851^**^ | -0.578^**^ | 0.864^**^ | 1.000 |
| **. Correlation is significant at the 0.01 level (2-tailed). | | | | | | | | |
| *. Correlation is significant at the 0.05 level (2-tailed). | | | | | | | | |

**Table S5** Correlation coefficient (r_s_) matrix demonstrating the relationship between estuarine sediment grain size (%) and organic matter content (%) (n=21).

|  | *E. coli* | Total Coliforms | *Salmonella* spp. | Enterococci | *Vibrio* spp. | | Organic matter |
| --- | --- | --- | --- | --- | --- | --- | --- |
| *E. coli* | 1.000 |  |  |  |  | |  |
| Total coliforms | 0.945^**^ | 1.000 |  |  |  | |  |
| *Salmonella* spp. | 0.763^**^ | 0.759^**^ | 1.000 |  |  | |  |
| Enterococci | 0.817^**^ | 0.780^**^ | 0.729^**^ | 1.000 |  | |  |
| *Vibrio* spp. | 0.847^**^ | 0.859^**^ | 0.709^**^ | 0.817^**^ | 1.000 | |  |
| Organic matter | 0.594^**^ | 0.545^*^ | 0.497^*^ | 0.765^**^ | 0.712^**^ | | 1.000 |
| **. Correlation is significant at the 0.01 level (2-tailed). | | | | | |  |  |
| *. Correlation is significant at the 0.05 level (2-tailed). | | | | | |  |  |

**Table S6** Correlation coefficient (r_s_) matrix demonstrating the relationship between the abundance of each cultured bacterial group within estuarine sediments and sediment organic matter content (n=21).

|  | *E. coli* | Total coliforms | *Salmonella* spp. | Enterococci | *Vibrio* spp. | Salinity | Temperature | Depth |
| --- | --- | --- | --- | --- | --- | --- | --- | --- |
| *E. coli* | 1.000 |  |  |  |  |  |  |  |
| Total coliforms | 0.945^**^ | 1.000 |  |  |  |  |  |  |
| *Salmonella* spp. | 0.763^**^ | 0.759^**^ | 1.000 |  |  |  |  |  |
| Enterococci | 0.817^**^ | 0.780^**^ | 0.729^**^ | 1.000 |  |  |  |  |
| *Vibrio* spp. | 0.847^**^ | 0.859^**^ | 0.709^**^ | 0.817^**^ | 1.000 |  |  |  |
| Salinity | -0.002 | -0.106 | -0.042 | -0.049 | -0.175 | 1.000 |  |  |
| Temperature | -0.129 | -0.014 | 0.077 | -0.048 | 0.084 | -0.884^**^ | 1.000 |  |
| Depth | 0.253 | 0.170 | 0.288 | 0.165 | 0.098 | 0.569^**^ | -0.353 | 1.000 |
| **. Correlation is significant at the 0.01 level (2-tailed). | | | | | | | | |

**Table S7** Correlation coefficient (r_s_) matrix demonstrating the relationship between the abundance of each cultured bacterial group within estuarine sediments and physico-chemical parameters measured directly above the bottom sediments (n=2).

|  | *E. coli* | Total coliforms | *Vibrio* spp. | Salinity | Temperature | Depth |
| --- | --- | --- | --- | --- | --- | --- |
| *E. coli* | 1.000 |  |  |  |  |  |
| Total coliforms | 0.808^**^ | 1.000 |  |  |  |  |
| *Vibrio* spp. | 0.474^*^ | 0.349 | 1.000 |  |  |  |
| Salinity | -0.294 | -0.336 | 0.544^*^ | 1.000 |  |  |
| Temperature | -0.164 | -0.194 | -0.614^**^ | -0.596^**^ | 1.000 |  |
| Depth | -0.332 | -0.403 | 0.091 | 0.511^*^ | -0.073 | 1.000 |
| **. Correlation is significant at the 0.01 level (2-tailed). | | | | | | |
| *. Correlation is significant at the 0.05 level (2-tailed). | | | | | | |

**Table S8** Correlation coefficient (r_s_) matrix demonstrating the relationship between the abundance of each cultured bacterial group within estuarine water and physico-chemical parameters measured at 0.2 m depth (n=21

| Bacterial group | Media | Incubation period |
| --- | --- | --- |
| *E.coli* / Coliforms* | Harlequin (LabM HAL008) | 24 h 37 ̊C |
| *Salmonella*  spp. | Harlequin Salmonella ABC Medium (LabM HAL001) | 24 h 37 ̊C |
| *Enterococci*  spp. | Slanetz & Bartley Medium (LabM LAB166) | 48 h 37 ̊C |
| *Campylobacter* spp. | Campylobacter Blood-free Selective Medium (LabM LAB112) supplemented with cefoperazone and amphotericin (LabM X112) | 48 h 37 ̊C under microaerophilic conditions using Campygen sachets (Oxoid, CN0035) |
| Total heterotrophs | Marine Agar (Deben Diagnostics Ltd, Ipswich UK) | 24 h Room temperature |
| *Vibrio*  spp. | Cholera Medium TCBS (Oxoid Ltd, Basingstoke, UK) | 24 h Room temperature |

**Table S9** Selective media used to enumerate target bacterial groups.
