# Supplementary material for: Sediment Composition Influences Spatial Variation in the Abundance of Human Pathogen Indicator Bacteria within an Estuarine Environment
Source: PLoS One. 2014 Nov 14;9(11):e112951. doi: 10.1371/journal.pone.0112951 (PMC4232572; doi:10.1371/journal.pone.0112951)
Supplement: Table S7 — Correlation coefficient (rs) matrix demonstrating the relationship between the abundance of each cultured bacterial group within estuarine sediments and sediment grain size (n = 21). (DOCX) [file pone.0112951.s007.docx]

**Table S7.** Correlation coefficient (r_s_) matrix demonstrating the relationship between the abundance of each cultured bacterial group within estuarine sediments and sediment grain size (n=21).

|  | *E. coli* | Total coliforms | *Salmonella* spp. | Enterococci | *Vibrio* spp. | Clay  (<4 µm) | Silt  (4 µm-63 µm) | Very fine sand (63 µm-125 µm) | Fine sand  (125 µm-250 µm) | Medium sand  (250 µm-500 µm) | Coarse sand  (500 µm-1000 µm) | Very coarse Sand  (1000 µm-2000 µm) |
| --- | --- | --- | --- | --- | --- | --- | --- | --- | --- | --- | --- | --- |
| *E. coli* | 1.000 |  |  |  |  |  |  |  |  |  |  |  |
| Total coliforms | 0.945^**^ | 1.000 |  |  |  |  |  |  |  |  |  |  |
| *Salmonella* spp. | 0.763^**^ | 0.759^**^ | 1.000 |  |  |  |  |  |  |  |  |  |
| Enterococci | 0.817^**^ | 0.780^**^ | 0.729^**^ | 1.000 |  |  |  |  |  |  |  |  |
| *Vibrio* spp. | 0.847^**^ | 0.859^**^ | 0.709^**^ | 0.817^**^ | 1.000 |  |  |  |  |  |  |  |
| Clay | 0.543^*^ | 0.495^*^ | 0.568^**^ | 0.664^**^ | 0.663^**^ | 1.000 |  |  |  |  |  |  |
| Silt | 0.570^**^ | 0.547^*^ | 0.578^**^ | 0.687^**^ | 0.688^**^ | 0.958^**^ | 1.000 |  |  |  |  |  |
| Very fine sand | 0.432 | 0.446^*^ | 0.368 | 0.625^**^ | 0.605^**^ | 0.804^**^ | 0.828^**^ | 1.000 |  |  |  |  |
| Fine sand | -0.457^*^ | -0.403 | -0.462^*^ | -0.618^**^ | -0.504^*^ | -0.795^**^ | -0.840^**^ | -0.539^*^ | 1.000 |  |  |  |
| Medium sand | -0.450^*^ | -0.416 | -0.419 | -0.650^**^ | -0.648^**^ | -0.899^**^ | -0.923^**^ | -0.935^**^ | 0.668^**^ | 1.000 |  |  |
| Coarse sand | 0.334 | 0.223 | 0.361 | 0.448^*^ | 0.266 | 0.486^*^ | 0.517^*^ | 0.214 | -0.774^**^ | -0.292 | 1.000 |  |
| Very coarse sand | 0.443^*^ | 0.353 | 0.398 | 0.634^**^ | 0.504^*^ | 0.625^**^ | 0.696^**^ | 0.461^*^ | -0.851^**^ | -0.578^**^ | 0.864^**^ | 1.000 |
| **. Correlation is significant at the 0.01 level (2-tailed). | | | | | | | | | | | | |
| *. Correlation is significant at the 0.05 level (2-tailed). | | | | | | | | | | | | |
