# Supplementary material for: Sediment Composition Influences Spatial Variation in the Abundance of Human Pathogen Indicator Bacteria within an Estuarine Environment
Source: PLoS One. 2014 Nov 14;9(11):e112951. doi: 10.1371/journal.pone.0112951 (PMC4232572; doi:10.1371/journal.pone.0112951)
Supplement: Table S8 — Correlation coefficient (rs) matrix demonstrating the relationship between estuarine sediment grain size (%) and organic matter content (%) (n = 21). (DOCX) [file pone.0112951.s008.docx]

**Table S8.** Correlation coefficient (r_s_) matrix demonstrating the relationship between estuarine sediment grain size (%) and organic matter content (%) (n=21).

|  | Organic  matter | Clay | Silt | Very fine  sand | Fine  sand | Medium  sand | Coarse  sand | Very coarse  sand |
| --- | --- | --- | --- | --- | --- | --- | --- | --- |
| Organic matter | 1.000 |  |  |  |  |  |  |  |
| Clay | 0.917^**^ | 1.000 |  |  |  |  |  |  |
| Silt | 0.926^**^ | 0.958^**^ | 1.000 |  |  |  |  |  |
| Very fine sand | 0.810^**^ | 0.804^**^ | 0.828^**^ | 1.000 |  |  |  |  |
| Fine sand | -0.834^**^ | -0.795^**^ | -0.840^**^ | -0.539^*^ | 1.000 |  |  |  |
| Medium sand | -0.903^**^ | -0.899^**^ | -0.923^**^ | -0.935^**^ | 0.668^**^ | 1.000 |  |  |
| Coarse sand | 0.527^*^ | 0.486^*^ | 0.517^*^ | 0.214 | -0.774^**^ | -0.292 | 1.000 |  |
| Very coarse sand | 0.736^**^ | 0.625^**^ | 0.696^**^ | 0.461^*^ | -0.851^**^ | -0.578^**^ | 0.864^**^ | 1.000 |
| **. Correlation is significant at the 0.01 level (2-tailed). | | | | | | | | |
| *. Correlation is significant at the 0.05 level (2-tailed). | | | | | | | | |
