# Supplementary material for: Sediment Composition Influences Spatial Variation in the Abundance of Human Pathogen Indicator Bacteria within an Estuarine Environment
Source: PLoS One. 2014 Nov 14;9(11):e112951. doi: 10.1371/journal.pone.0112951 (PMC4232572; doi:10.1371/journal.pone.0112951)
Supplement: Table S9 — Correlation coefficient (rs) matrix demonstrating the relationship between the abundance of each cultured bacterial group within estuarine sediments and sediment organic matter content (n = 21). (DOCX) [file pone.0112951.s009.docx]

**Table S9.** Correlation coefficient (r_s_) matrix demonstrating the relationship between the abundance of each cultured bacterial group within estuarine sediments and sediment organic matter content (n=21).

|  | *E. coli* | Total Coliforms | *Salmonella* spp. | Enterococci | *Vibrio* spp. | | Organic matter  content |
| --- | --- | --- | --- | --- | --- | --- | --- |
| *E. coli* | 1.000 |  |  |  |  | |  |
| Total coliforms | 0.945^**^ | 1.000 |  |  |  | |  |
| *Salmonella* spp. | 0.763^**^ | 0.759^**^ | 1.000 |  |  | |  |
| Enterococci | 0.817^**^ | 0.780^**^ | 0.729^**^ | 1.000 |  | |  |
| *Vibrio* spp. | 0.847^**^ | 0.859^**^ | 0.709^**^ | 0.817^**^ | 1.000 | |  |
| Organic matter | 0.594^**^ | 0.545^*^ | 0.497^*^ | 0.765^**^ | 0.712^**^ | | 1.000 |
| **. Correlation is significant at the 0.01 level (2-tailed). | | | | | |  |  |
| *. Correlation is significant at the 0.05 level (2-tailed). | | | | | |  |  |
