# Supplementary material for: Sediment Composition Influences Spatial Variation in the Abundance of Human Pathogen Indicator Bacteria within an Estuarine Environment
Source: PLoS One. 2014 Nov 14;9(11):e112951. doi: 10.1371/journal.pone.0112951 (PMC4232572; doi:10.1371/journal.pone.0112951)
Supplement: Table S10 — Correlation coefficient (rs) matrix demonstrating the relationship between the abundance of each cultured bacterial group within estuarine sediments and physico-chemical parameters measured directly above the bottom sediments (n = 2). (DOCX) [file pone.0112951.s010.docx]

**Table 10.** Correlation coefficient (r_s_) matrix demonstrating the relationship between the abundance of each cultured bacterial group within estuarine sediments and physico-chemical parameters measured directly above the bottom sediments (n=2).

|  | *E. coli* | Total coliforms | *Salmonella* spp. | Enterococci | *Vibrio* spp. | Salinity | Temperature | Depth |
| --- | --- | --- | --- | --- | --- | --- | --- | --- |
| *E. coli* | 1.000 |  |  |  |  |  |  |  |
| Total coliforms | 0.945^**^ | 1.000 |  |  |  |  |  |  |
| *Salmonella* spp. | 0.763^**^ | 0.759^**^ | 1.000 |  |  |  |  |  |
| Enterococci | 0.817^**^ | 0.780^**^ | 0.729^**^ | 1.000 |  |  |  |  |
| *Vibrio* spp. | 0.847^**^ | 0.859^**^ | 0.709^**^ | 0.817^**^ | 1.000 |  |  |  |
| Salinity | -0.002 | -0.106 | -0.042 | -0.049 | -0.175 | 1.000 |  |  |
| Temperature | -0.129 | -0.014 | 0.077 | -0.048 | 0.084 | -0.884^**^ | 1.000 |  |
| Depth | 0.253 | 0.170 | 0.288 | 0.165 | 0.098 | 0.569^**^ | -0.353 | 1.000 |
| **. Correlation is significant at the 0.01 level (2-tailed). | | | | | | | | |
