# Supplementary material for: Sediment Composition Influences Spatial Variation in the Abundance of Human Pathogen Indicator Bacteria within an Estuarine Environment
Source: PLoS One. 2014 Nov 14;9(11):e112951. doi: 10.1371/journal.pone.0112951 (PMC4232572; doi:10.1371/journal.pone.0112951)
Supplement: Table S11 — Correlation coefficient (rs) matrix demonstrating the relationship between the abundance of each cultured bacterial group within estuarine water and physico-chemical parameters measured at 0.2 m depth (n = 21). (DOCX) [file pone.0112951.s011.docx]

**Table S11.** Correlation coefficient (r_s_) matrix demonstrating the relationship between the abundance of each cultured bacterial group within estuarine water and physico-chemical parameters measured at 0.2 m depth (n=21).

|  | *E. coli* | Total coliforms | *Vibrio* spp. | Salinity | Temperature | Depth |
| --- | --- | --- | --- | --- | --- | --- |
| *E. coli* | 1.000 |  |  |  |  |  |
| Total coliforms | 0.808^**^ | 1.000 |  |  |  |  |
| *Vibrio* spp. | 0.474^*^ | 0.349 | 1.000 |  |  |  |
| Salinity | -0.294 | -0.336 | 0.544^*^ | 1.000 |  |  |
| Temperature | -0.164 | -0.194 | -0.614^**^ | -0.596^**^ | 1.000 |  |
| Depth | -0.332 | -0.403 | 0.091 | 0.511^*^ | -0.073 | 1.000 |
| **. Correlation is significant at the 0.01 level (2-tailed). | | | | | | |
| *. Correlation is significant at the 0.05 level (2-tailed). | | | | | | |
